# Acoustic Wave Sensors for Detection of Blister Chemical Warfare Agents and Their Simulants

**DOI:** 10.3390/s22155607

**Published:** 2022-07-27

**Authors:** Michał Grabka, Zygfryd Witkiewicz, Krzysztof Jasek, Krzysztof Piwowarski

**Affiliations:** Institute of Chemistry, Faculty of Advanced Technologies and Chemistry, Military University of Technology, 00-908 Warsaw, Poland; zygfryd.witkiewicz@wat.edu.pl (Z.W.); krzysztof.jasek@wat.edu.pl (K.J.); krzysztof.piwowarski@wat.edu.pl (K.P.)

**Keywords:** SAW, QCM, sulfur mustard, blister agent detection, CEES

## Abstract

On-site detection and initial identification of chemical warfare agents (CWAs) remain difficult despite the many available devices designed for this type of analysis. Devices using well-established analytical techniques such as ion mobility spectrometry, gas chromatography coupled with mass spectrometry, or flame photometry, in addition to unquestionable advantages, also have some limitations (complexity, high unit cost, lack of selectivity). One of the emerging techniques of CWA detection is based on acoustic wave sensors, among which surface acoustic wave (SAW) devices and quartz crystal microbalances (QCM) are of particular importance. These devices allow for the construction of undemanding and affordable gas sensors whose selectivity, sensitivity, and other metrological parameters can be tailored by application of particular coating material. This review article presents the current state of knowledge and achievements in the field of SAW and QCM-based gas sensors used for the detection of blister agents as well as simulants of these substances. The scope of the review covers the detection of blister agents and their simulants only, as in the available literature no similar paper was found, in contrast to the detection of nerve agents. The article includes description of the principles of operation of acoustic wave sensors, a critical review of individual studies and solutions, and discusses development prospects of this analytical technique in the field of blister agent detection.

## 1. Introduction

The 1993 Chemical Weapons Convention (CWC) completely prohibits the use of chemical weapons and the research into new substances, and mandates the destruction of their stockpiles [1]. To date, the CWC has been signed and ratified by 193 countries, with approximately 98% of the world’s population [2]. Due to the still substantial existing chemical weapon (CW) stocks, ease of production, and the fact that not all countries have signed and ratified the CWC, the use of chemical weapons in the modern battlefield or during terrorist attacks should still be taken into account.

In the case of the use of CWA, fast detection and initial identification are particularly important [3,4,5,6]. Various methods and analytical procedures are used for this purpose. The most important methods of CWA analysis include chromatographic techniques combined with mass spectrometry [7,8,9,10]. The results of analyses carried out with such devices in stationary laboratories usually leave no room for doubt. For field analysis, mobile and portable gas chromatographs combined with mass spectrometers (GC-MS) are very useful [11,12,13,14]. The use of mobile analytical instruments follows the general trend of performing on-site analyses. This approach minimizes the problems related to the sample stability and contamination of the sample during transport. Moreover, it allows to shorten the time from sampling to result, which is especially important in the case of highly toxic substances.

Portable GC-MS enables the detection and identification of CWA with high accuracy [15]. However, these devices are still expensive and require well-trained staff. They are also relatively heavy and require efficient power sources. Therefore, research is still being conducted on other portable devices that would have slightly worse analytical properties than GC-MS, and at the same time would meet the requirements for the analysis of highly toxic substances. In particular, these devices should enable the detection of CWAs at concentrations below the toxic threshold concentrations of these substances and enable the initial identification. Such devices should be lightweight, easy to use, and cheap. Moreover, its dimensions and the possibility of operation automation should enable their use in platforms for remote CW reconnaissance, e.g., in unmanned aerial vehicles.

Sensors using various analytical techniques are used for on-site CWA detection. Among them are devices using well-established techniques such as ion mobility spectrometry (IMS) [16] and flame photometry (FP) [17] but also analytical techniques based on simple and undemanding sensors. Recently, semiconductor [18,19], colorimetric, and fluorescent [20,21] sensors have also attracted a lot of interest. Especially fluorescent/colorimetric sensors have a good chance of being used in on-site analyzers. In addition to portable analyzers, these sensors can be used in contamination indicators and chemical dosimeters in line with the idea of wearable detectors. Furthermore, the wide possibilities of adjusting the selectivity of fluorescent and colorimetric sensors allow them to be used not only in military applications, but also in agriculture and the food industry [22,23].

In addition to the above-mentioned techniques, acoustic wave sensors are used for on-site CWA detection [24]. As acoustic wave gas sensors meet all requirements for mobility (light weight, low energy consumption) and affordability (low cost, ease of use) well, they are seen as a potential technique for detecting CWA and other hazardous substances. Despite the fact that acoustic wave chemical gas sensors are not a very new issue (the first work on gas sensors with quartz crystal microbalance (QCM) dates from 1964 [25] while the first sensor with a surface acoustic wave (SAW) device was described in 1979 [26]), work on these devices is still very intensive as evidenced by the number of publications in scientific journals in recent years.

In 2017, a comprehensive review was published on the detection of volatile organic compounds (VOCs) and other gases with SAW sensors [27]. Another review from 2022 [28] presents achievements in the field of gas sensors based on different types of acoustic wave devices, including QCM. The works cited summarize the achievements in the field of acoustic wave gas sensors for the detection of vapors of various organic compounds and gases. The available scientific literature also includes studies focused solely on the detection of nerve CWAs [29]. However, there is a lack of work devoted to the detection of another group of CWAs of great military importance—blister agents.

In this review, we describe the state of the art and achievements in the field of detection of blister agents (mainly sulfur mustard) using SAW and QCM-based gas sensors. Due to the high toxicity of blister CWAs and the strict regulation of Organization for the Prohibition of Chemical Weapons, most studies concern the detection of simulants of these substances, which are characterized by a similar structure and physicochemical properties, but much lower toxicity [30].

## 2. Surface Acoustic Wave and Quartz Crystal Microbalance Devices

The group of acoustic wave sensors includes several types of piezoelectric devices, among which we can list quartz crystal microbalances, a variety of surface acoustic wave sensors, and other sensors such as polymorphic sensors with different geometries (cantilever, fork, disk-shaped etc.) [31,32]. Sensors of this type are characterized by a very small size, uncomplicated structure, low power consumption, and simple electronic systems, which makes them an ideal candidate for use in portable instruments, in particular for the detection of CWAs and toxic industrial chemicals (TICs) in the field.

**Figure 1:**
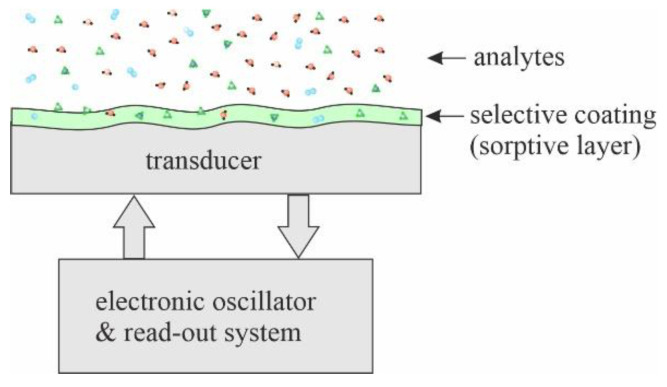
The basic principle of operation of an acoustic wave chemical gas sensor.

Regardless of the type of piezoelectric device used, each sensor consists of a transducer with an acoustic wave and a selective coating that interacts with the analyte present in the environment. As a result of this interaction, the parameters of the coating, such as mass, density, stiffness, and electrical conductivity, change. These changes affect the propagation of the acoustic wave in the transducer material to which the sorption layer adheres, changing its speed and attenuation. An ordinary piezoelectric transducer is equipped with a resonant system, which vibrates with a strictly defined frequency depending on the parameters of the acoustic wave. Associated electronic systems convert acoustic wave parameters into electrical quantities. As a result, changes in wave parameters caused by analyte sorption are observed as changes in the resonant frequency, phase, and attenuation in the resonance system. A general diagram of the operation of an acoustic wave chemical gas sensor is shown in Figure 1.

**Figure 2:**
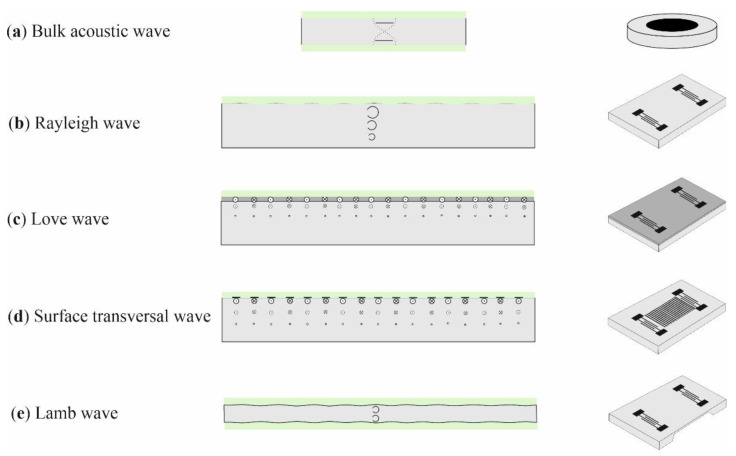
Wave propagation modes and acoustic wave transducers used in chemical gas sensors.

There are many types of acoustic wave transducers differing in the type of acoustic wave propagating and the method of its excitation. The most widely used in gas sensing applications are transducers with bulk acoustic wave, Rayleigh and Love-type surface acoustic waves, and plate waves [33]. A general scheme of the construction of these transducers and the wave propagating modes are shown in Figure 2.

The bulk wave transducers are in the form of a plate made of piezoelectric material with metallic electrodes applied on both sides (Figure 2a). Usually this material is AT-cut quartz, therefore such sensors are called quartz crystal microbalance or quartz microbalance (QMB). Vibrations, excited in the crystal, have the form of a shear, hence the name TSM (transverse shear mode) sensors can also be found.

Another type of transducer is a group of SAW devices. In this case, the wave is excited by interdigital electrodes applied on the surface of a piezoelectric substrate. This group includes transducers both with particle displacement oriented perpendicularly (Rayleigh wave, Figure 2b) and parallel (Love wave and surface transversal wave—STW, Figure 2c,d) to the plane of the substrate coated with the sorptive layer. The Love-type SAW propagates within an additional, thin layer of solid material (guiding layer) applied on the piezoelectric substrate. In turn, STW propagates in the very substrate. Both Love-type SAW and STW can also be used for measurements in a liquid medium due to much lower attenuation than in the case of Rayleigh wave.

The third group consists of plate transducers, in which the wave propagates in a thin membrane (Lamb wave, Figure 2e).

In addition to the acoustic wave transducers presented in Figure 2, there are also many other transducer geometries and types of acoustic waves [32]; however, those listed here constitute the vast majority of structures used in chemical gas sensors.

Individual acoustic wave transducers differ in the range of operating frequencies, the ability to work in various environments (liquid and gas), sensitivity to changes in the parameters of the selective coating, and many other properties. A comprehensive description of their operation and application can be found, among others in works [32,34,35]. However, regardless of the type of transducer used, the selective coating is a key component of any chemical sensor of this type. It determines the basic parameters of the sensor, such as its sensitivity, selectivity, durability, reversibility, chemical resistance, and dynamic properties.

## 3. Blister Agents and Their Simulants

Groups of blister agents (also known as vesicant) include sulfur mustards (code name: HD), nitrogen mustards, and lewisites. These substances are listed on Schedule A of the CWC. In research on CWAs on-site detection techniques, simulants of these compounds are also often used.

For historical reasons (large scale use during World War I), the best-known blister agent is sulfur mustard, which is perceived by the public as particularly dangerous. Main symptoms of sulfur mustard intoxication are chemical burns of the skin and mucous membranes. In addition, it shows strong mutagenic effects. The IDLH concentration (immediately dangerous to life or health) of this compound in the air is 0.7 mg·m^−3^ [36]. Furthermore, sulfur mustard in a liquid and solid state (solidification point around 14 °C) is very dangerous when it comes into contact with the skin and mucous membranes. In addition to the danger associated with the use during military conflicts, the incidents of finding ammunition filled with sulfur mustard during earthworks or caught in fishing nets (significant amounts of these weapons were dumped in the Baltic Sea after World War II) also pose a great risk [37].

**Figure 3:**
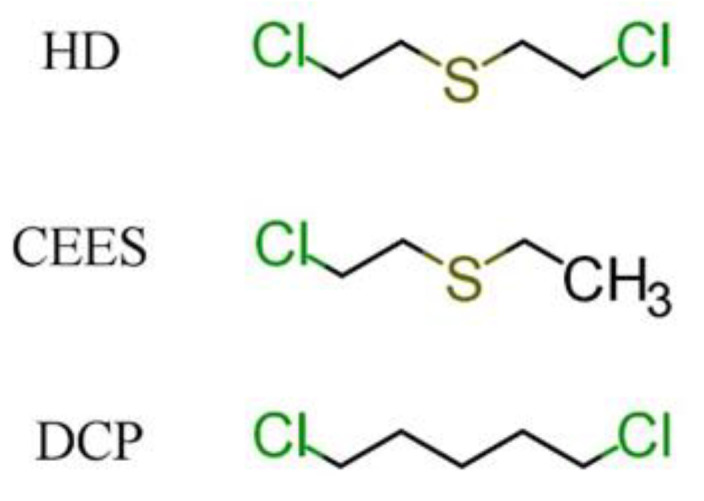
Sulfur mustard (HD) and its simulants: 2-chloroethyl ethyl sulfide (CEES) and 1,5-dichloropentane (DCP).

Due to the reasons set out above, most of the research in the field of blister CWAs detection, including work on acoustic wave sensors, focuses on the detection of sulfur mustard and its simulants. Figure 3 shows the structures of the sulfur mustard and its two most frequently used simulants: 2-chloroethyl ethyl sulfide (CEES) and 1,5-dichloropentane (DCP).

Table 1 shows some of the physicochemical properties of these compounds and the toxicity data.

Apart from the substances listed above, other simulants of sulfur mustard were also used in the research on acoustic wave sensors: 1,2-dichloroethane (DCE), dichloroethyl ether (DCEE), dibuthyl sulfide (DBS), chloroethyl phenyl sulfide (CEPS), methyl salicylate (MS), and dimethylacetamide (DMA). A nitrogen mustard simulant—dipropylene glycol monomethyl ether (DPGME) was also used in some studies. It is noteworthy that some of the simulants mentioned here show a significant difference in the structure and physicochemical properties compared to the actual CWA.

In the case of acoustic wave sensors, the selection of simulants for specific tests is related to the type of selective coating used. Depending on what properties of the target analyte are used for its detection, individual simulants should show similarity in the area of these features. For example, when target analyte detection occurs through its absorption in the coating, the simulant should be characterized by similar solvation properties. On the other hand, when the distinction is made by means of differences in volatility, the simulant should have a similar volatility as actual CWA, etc.

## 4. Detection of Blister Agents and Their Simulants Using Acoustic Wave Sensors

The basic metrological parameters of any sensor to evaluate its suitability for a particular application are limit of detection (LOD), limit of quantification (LOQ), and selectivity. In the case of acoustic wave sensors, all these parameters are mostly influenced by the selective coating used. The improvement of the metrological parameters of the sensor can be achieved by using sorptive layers tailored to the analyte being detected. Another approach is to use an array of sensors in which each sensor is equipped with a sorptive layer with different properties. In this case, the analytical information is contained in the multivariate response of the sensor array. Analyzing the available literature, it can be concluded that both approaches are used in the development of sensors for the detection of blister CWAs. Due to the fact that the main distinguishing feature of the sensors being the subject of this review is the type of the selective coating, the work was divided into sub-chapters using this criterion.

### 4.1. Sensor with Layers of Non-Conductive Polymers at the Elastomeric State

The most commonly used type of selective coating in acoustic wave chemical gas sensors are absorption layers made of non-conductive elastomers. In that case, the analyte is physically absorbed from the gas phase in the liquid polymer (this process is equated with dissolution, where the polymer is a solvent and the analyte is a dissolved substance). As a result, the mass of the layer and its elasticity parameters (shear and bulk moduli) change. These changes affect the acoustic wave propagating in the substrate under the sorptive coating, leading to a measurable change in the frequency or amplitude of vibrations.

The distribution of the analyte present in the ambient gas between the gas phase and the sorptive layer at the thermodynamic equilibrium is described by Henry’s law [41]:

(1) $$K=\frac{C_{S}}{C_{V}},$$

where:

**K** partition constant,

**C_s_** equilibrium analyte concentration in the sensor layer,

**C_v_** equilibrium analyte concentration in ambient gas.

The acoustic wave sensor response is proportional to the amount of analyte sorbed in the sensor layer according to the following general equation [42]:

(2) $$response=S\cdot C_{V}\cdot K,$$

where:

**S** sensitivity factor as a function of analyte specific factors, i.e., vapor-specific volume, and sensor/transducer related factors, i.e., type of acoustic wave device used, center frequency etc.

It can be seen from the Equation (2) that the higher the **K** value, the higher be the sensor response value for a given analyte concentration. In order for the sensor to be selective, only a specific chemical individual or family of chemical compounds should have a high **K** value.

In the case of absorption layers, sorption of the analyte in the polymer is the result of non-covalent intermolecular interactions occurring in the liquid solution, such as induced dipole–induced dipole (Van der Waals forces), constant dipole–constant dipole, constant dipole–induced dipole, or formation of hydrogen bonds. The **K** value, and thus the response value and the selectivity of the sensor, can be influenced by adjusting the solvation properties of the sensor layer to the analyte, e.g., by introducing certain functional groups into the polymer chain.

In the case of elastomeric absorption layers, various solvation models are used to describe the sorption mechanism [43]. One of the most relevant is the linear solvation energy relationship—LSER. This model quantitatively characterizes the solvation properties of the solute (analyte) and solvent (polymer) by means of a set of coefficients and allows predicting the value of the partition constant of the analyte in a given system. Expression for the value of the partition constant of the analyte in the liquid–gas system (which corresponds to the operating conditions of the gas sensor) can be written as [44]:

(3) logK=c+eE+sS+aA+bB+lL,

In Equation (3), the coefficients marked with capital letters describe the properties of the solute: **E** is the calculated excess molar refractivity relative to an n-alkane with the same McGowan volume; **S** describes solute dipolarity/polarizability; **A** and **B** are measures of hydrogen bond acidity and basicity; **L** is the decimal logarithm of the gas to n-hexadecane partition coefficient measured at 298 K. The coefficients marked with lowercase letters describe the complementary properties of the solvent, while c is a system constant, and it results from the mathematical method of determining the set of parameters.

According to Equation (3), the total sorption is the sum of the contributions from individual intermolecular interactions occurring in the solution between the polymer and the analyte. The shares of individual types of interactions are the products of the values of the complementary LSER coefficients for the polymer and the analyte. For example, to calculate the sorption by acid hydrogen bonds formation by the polymer, multiply the b-value of the polymer and the **B**-value of the analyte, etc.

Solvation models are used in many aspects. Apart from the description of the sorption mechanism, the coefficients may be a criterion for the selection of polymers for the sensor arrays [45,46,47,48,49]. Solvation models are also used to identify unknown substances based on the response of the sensor array [50].

Despite the possibility of adjusting the solvation properties of the polymers, e.g., by structural modifications, a single sensor with an absorption layer has a rather limited selectivity. For this reason, the dominant part of the published works describes sensor arrays with layers of this type. Sensor arrays, also known as electronic noses (e-noses), consist of a group of sensors, each of which is equipped with a coating with different solvation properties. The responses of the individual sensors correspond to the amount of analyte absorbed in its layer. This in turn depends on the complementarity of the solvation interactions of the analyte and the coating material. Detection of an analyte results in a set of response values of individual sensors. In some simplification, such a set can be treated as specific for a given analyte or group of analytes.

Numerical processing of this type of data, with the use of appropriate algorithms, makes it possible to identify or at least classify detected substances. Currently, many algorithms are used to interpret the array response. Among the most commonly used are dimension reduction algorithms, e.g., principal component analysis (PCA), and pattern recognition algorithms, e.g., linear classifiers, neural networks (NN) and neighborhood approaches. Empirical comparisons of individual numerical methods in terms of their practical application to the array response analysis are presented in the works [51,52]. The criteria for the algorithms’ comparison at work [51] were, among others, accuracy, speed of operation, speed and ease of learning, low memory requirements, and non-susceptibility to outliers. Based on the performed calculations, it was found that the neural networks algorithms are the most suitable for use in sensor arrays. These methods allow to obtain high rate of correctly classified patterns and at the same time are possible to implement with the use of limited computing power of microprocessors, e.g., in portable devices. The good properties of neural networks have also been confirmed in more recent work [52]. In this paper, the large margin nearest neighbors algorithm was also indicated as very useful. Moreover, the study indicated that the use of PCA in the process of data preparation for the algorithm using NN significantly improves classification performance.

In some works [47,48,53], the responses of the arrays are presented in radial charts constituting the fingerprint of individual analytes. These types of charts allow for the visual differentiation of particular substances.

**Figure 4:**
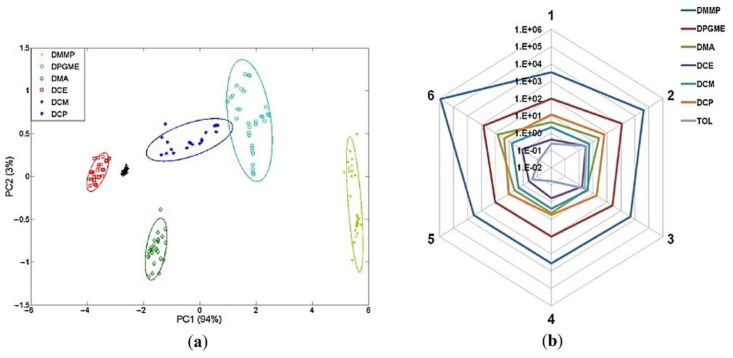
Some methods of interpreting the response of sensor array: (**a**) Principal Component Analysis (PCA) obtained for the matrix of six Love wave sensors. In this case, the PCA made it possible to clearly distinguish six compounds that are simulants of sulfur mustard, nitrogen mustard, and sarin. (**b**) The radial plot of array response. Each axis represents an individual sensor and the distance from the center of the plot represents the sensitivity expressed in Hz·ppm^−1^. These graphs represent the fingerprints of the individual analytes and can be used for their visual differentiation [48]. Reprinted with permission from [48]. Copyright 2011 Elsevier.

Figure 4 shows the results of some methods of interpreting the response of sensor arrays.

Typically, the sensor array includes a single sensor that exhibits a particularly high sensitivity to target analyte. The analysis of the available literature indicates that sensors with poly(epichlorohydrin)—PECH—are used for this purpose in case of detection of HD and its simulants. It can be said that PECH sensors are an inherent element of such arrays, which is confirmed by numerous scientific publications.

**Figure 5:**
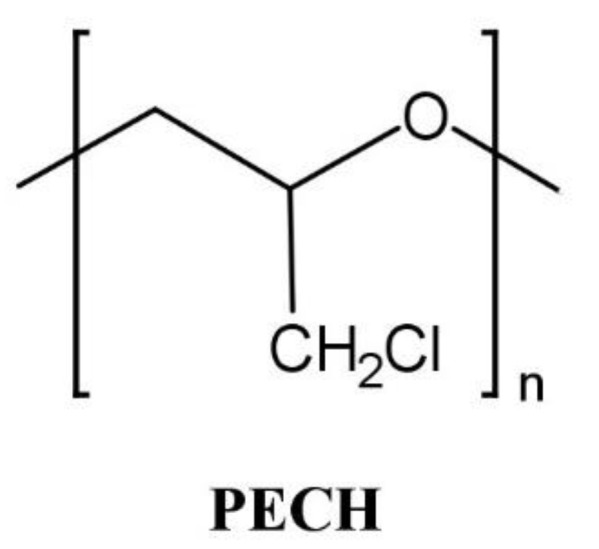
Monomer of poly(epichlorohydrin)—PECH.

PECH is a functional polyether obtained by catalytic ring-opening polymerization of epichlorohydrin. In addition to its applications in sensors, PECH is widely used in industry [54]. Figure 5 shows the structure of PECH.

The PECH glass transition temperature (T_g_) values reported in the literature range from −22.4 °C [55] to −14.5 °C [56]. Such low values of T_g_ confirm the definitely elastomeric nature of PECH at typical operating temperatures of SAW sensors.

The solvation properties of PECH are determined by dipolar chloromethyl groups and slightly basic ether linkages. The interaction of PECH with sulfur mustard is likely to be Van der Waals interactions and the formation of hydrogen bonds in which PECH is donor of hydrogen [55,57]. However, the contribution of formation of hydrogen bonds of this type has been questioned in some work [58]. The cited study investigated the response of a sensor equipped with elastomer with a strong and selective ability to form acid hydrogen bonds (PLF-polymethyl [3-(1,1,1,3,3,3-hexafluoropropan-2-ol)propyl]siloxane) to HD. In this case, only a slight sensitivity to HD was observed, which casts doubt on the significant share of this type of hydrogen bonding in HD sorption.

The LSER coefficients of the PECH have been published in the literature [59]. The values of the coefficients are presented in Table 2.

Based on these values, it can be said that PECH has rather non-specific solvation interactions. PECH is a polymer of considerable polarity, and it acts rather as an acceptor of the hydrogen atom during the formation of hydrogen bonds.

The first use of PECH in acoustic wave gas sensors was described in 1986 [60]. In the cited work, however, the PECH sensor was not used to detect blister agents, but as an element of the sensor array for the detection and identification of other organic vapors. In the following years [50,53,61], PECH sensors were used in sensor arrays to detect vapors of various substances, including nerve CWA simulants. However, also this time no blister CWAs or their simulants were detected with these arrays. The purpose of using PECH in these studies was to diversify the solvation properties of the arrays rather than to match the solvation properties to a specific analyte.

The first paper describing the detection of blister CWAs with the use of PECH sensor was published in 1993 [62]. The cited work describes a prototype system for the detection of organophosphorus CWAs and sulfur mustard, the key element of which was an array of four SAW sensors. As SAW devices, acoustic delay lines with a center frequency of 158 MHz were used. The resonators were spray-coated with four different elastomers (the thicknesses of the applied films were estimated at 40–80 nm). Apart from PECH, fluoropolyol (FPOL), poly(ethylenimine) (PEI), and ethyl cellulose (ECEL) were used [53]. The device was equipped with an automatic sampling system with preconcentrator (thermally desorbed sorption tubes with a 40–60 mesh Tenax GC). The investigation of the array responses showed that the PECH sensor had the highest response to sulfur mustard among the remaining sensors in the array. The system enabled the detection of sulfur mustard at a concentration of 10 mg·m^−3^ in the direct analysis mode and 0.5 mg·m^−3^ with a 2-min preconcentration. As the studies showed, it was possible to obtain lower detection limits by using a longer preconcentration time (with the use of 14-min preconcentration, HD was detected at a concentration of 0.05 mg·m^−3^). The study demonstrated that this technique of detection is effective and possible to implement in a single, portable device. The research proved that it is possible to detect sulfur mustard in concentrations lower than IDLH which is 0.7 mg·m^−3^ [37]. The paper emphasizes the role of preconcentration, thanks to which it was possible to significantly lower the CWAs detection limits and minimize the influence of interferents.

In 2007 [56], another sensor array designed for the detection of CWAs vapors was described. In this work, an array of five SAW sensors coated with PECH, polyisobutylene (PIB), polydimethylsiloxane (PMDS), polyisoprene (PIP), and polybutadiene (PDB) were used. The array was used to detect vapors of simulants of various CWAs groups. DCP was used as the simulant of sulfur mustard. The response of the array was analyzed using PCA. This made it possible to distinguish DCP from other simulants. Results showed that the PIP sensor had the highest sensitivity to DCP. In contrast, the sensor with PECH showed significantly lower sensitivity to this substance. The reason for this may be due to differences in the properties of DCP and HD. Although DCP shows some similarity in structure to HD, its solvation properties may be significantly different. The thesis of low suitability of DCP as a HD simulant was also put forward in [62].

**Figure 6:**
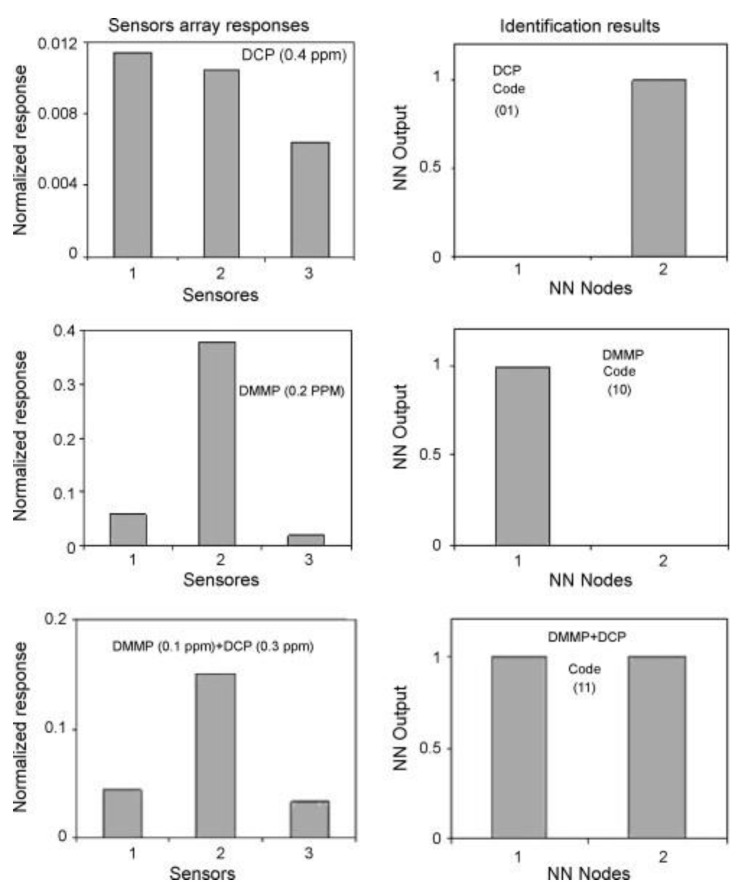
Examples of normalized responses of sensor array (sensor 1—SXFA, sensor 2—PECH, sensor 3—OV25) for given simulant (DCP, DMMP, mixture of DCP and DMMP) and ANN classification results [63]. Reprinted with permission from [63]. Copyright 2008 Elsevier.

Another sensor array with PECH was described in 2008 [63]. In this study, an array of three SAW sensors was used to distinguish vapors of sulfur mustard and G-series nerve CWAs simulants (DCP and DMMP). In this case, three different elastomers were used: PECH, SXFA (hexafluoro-2-propanol-substituted polysiloxane with high acid hydrogen bonding capacity), and OV25 (commercially available GC stationary phase, poly[(methylphenylsiloxane)-co-(diphenylsiloxane)] with 75% of phenyl groups and 25% of methyl groups, characterized by high polarity and polarizability). The selection of polymers for the array was based on the LSER model. The array was equipped with a preconcentrator (Tenax GR bed and thermal desorber). The paper presents the results of the analysis of DCP, DMMP, and their mixtures. As was shown, the array correctly detected and classified analytes, both individually and in a mixture, in concentrations equal to tenths of a ppm. The use of pattern recognition algorithms (artificial neural network (ANN) and PCA-ANN) led to more than 95% of correct classifications. The PECH sensor showed the highest sensitivity to DCP. At the same time, it showed some cross-sensitivity to DMMP. Thanks to the applied thermal desorber, it was possible to remove interferents, mainly water, from the gas fed to the array. The desorber program consisted of preheating to 55 °C (the first 10 s of the program) followed by heating to 250 °C. During preheating, volatile interferences, including water vapor, desorbed, and the gas flow was directed to the atmosphere bypassing the array. Examples of normalized array responses and classification results using ANN algorithm are shown in Figure 6.

Although the available literature is dominated by descriptions of sensor arrays, there are also studies of individual sensors for HD detection. The work [64] describes the detection of sulfur mustard vapors with a single sensor based on a two-port SAW resonator equipped with a PECH layer. The sensor with the center frequency of 200 MHz showed a sensitivity to HD of 106 Hz·mg^−1^·m^3^ and a limit of detection equal to 0.3 mg⋅m^−3^. Moreover, the response versus concentration curve was linear over the entire tested concentration range (from 1.2 to 61.7 mg·m^−3^). The sensor was also found to have good selectivity and to be significantly less sensitive to organophosphorus compounds than to HD.

In a series of works [45,46,47,48,49], the authors present their achievements in the field of optimization of acoustic wave sensor arrays for the detection and classification of blister and nerve CWAs. They focus on both Rayleigh wave [45,47] and Love wave SAW sensors [46,48,49]. In addition to PECH, their arrays usually utilize following elastomers: PEI, PDMS, polycyanopropylmethyl siloxane (PCPMS), Carbowax (CW), trifluoropropylmethyl siloxane-dimethylsiloxane (PMFTPMS). Both the selection of polymers and the determination of the array sizes were based on the LSER model. For the analysis of the array’s responses, the authors used various numerical methods. Among them we can find PCA, probabilistic neural network (PNN), and radial charts. In the works cited, authors used CWAs simulants. DCP, DPE, and DMA were used as sulfur mustard simulants, while as nitrogen mustard, DPGME. Based on the analysis of arrays responses, the authors draw conclusions about the effectiveness and usefulness of this analytical technique. Nevertheless, due to the significant differences between the simulants used in the works and the actual CWAs, the questions about the behavior of the sensor arrays in the case of actual CWAs detection reappear.

The development in the field of sensor arrays is taking place not only in the area of new coating materials and methods of arrays response analysis. Currently, there are also advanced application works related to the use of the acoustic wave sensor technique in various detection systems, e.g., wireless sensor network (WSN).

In the work from 2018 [65], the sensor array for the remote detection of CWAs and other toxic gases was described. The array was composed of four two-port SAW resonators coated with: PECH, SXFA, TEA (triethylamine), and L-glutamic acid hydrochloride (the film thicknesses ranged from 50 to 100 nm). In this study, vapors of CEES, DMMP, H_2_S, and NH_3_ were detected. The PECH sensor showed the highest sensitivity to CEES equal to 14.9 Hz ppm^−1^ and significant cross-sensitivity to H_2_S. Nevertheless, the analysis of the array responses allowed for an unambiguous distinction between these substances. The work puts a lot of emphasis on demonstrating the use of the SAW arrays as an element of the WSN. According to the idea of WSN, the SAW array was to be a sensor element of a scattered sensor network. A single array performs the analyses in a fully automatic manner and transmits the measurement results to the master station. The sensor array was therefore equipped with a wireless communication and GPS module. As demonstrated by field trials, it was possible to communicate with the arrays and collect data on detected substances in real time within a radius of 300 m from the master station.

Currently, research on the use of PECH in sensors for the detection blister agents is still being continued. Interesting results have been presented in the works that have appeared in recent years [54,57].

The work in [57] describes a PECH sensor using an SAW delay line with a center frequency of 150 MHz. This sensor was used to detect CEES vapors in the air. The CEES detection limit of 1.5 mg·m^−3^ and the sensitivity of 233.17 Hz·mg^−1^·m^3^ were achieved. The response versus concentration curve was linear in the range of 1.2–10 mg·m^−3^. The measurements revealed that the sensor has good short and long-term repeatability (the sensitivity of the sensor has decreased by approximately 16% over the 18 months). Moreover, the sensor showed a high selectivity to CEES, although some cross-sensitivities were found to organophosphorus pesticides, amines, and some organic acids. The study also investigated the influence of temperature and water vapor content on the PECH sensor performance. As could be expected, with the increase of temperature, the sensitivity of the sensor decreases (the CEES vapor solubility in the elastomer decreases) and its dynamic parameters improve (the reaction and regeneration time decreases as a result of increase in vapor diffusion rate in the elastomer). With the increase in gas humidity in the range of 30–80% relative humidity, a significant increase in sensitivity to CEES was observed.

In 2022, the same research team published another paper on the use of a PECH sensor to detect CEES [55]. This time, a SAW delay line with a center frequency of 200 MHz was used. The manufactured sensor was characterized by the target analyte detectability at the level of 0.85 mg·m^−3^ with the limit of quantification equal to 1.91 mg·m^−3^. Much attention in this paper was devoted to the investigation of the morphology and other parameters of the sorptive layers (the layers were applied using the spin-coating method).

In addition to PECH, the available literature presents other elastomeric materials for absorption layers. Interesting results are presented in the paper [66], in which for the detection of sulfur mustard simulants (DCEE) and nerve agents simulant (DMMP), a QCM sensor coated with poly(N,N-dimethylaminoethyl methacrylate)/polyvinyl alcohol copolymer (PDMAEMA/PVA) was used. The research revealed that the coating reversibly absorbs the HD simulant and is suitable for its detection. In addition, the sensor showed short response and regeneration time. In the case of nerve CWA simulants, irreversible chemical sorption took place. Moreover, it was found that in the case of diethyl chlorophosphate, a chemical reaction takes place, leading to the degradation of this compound (on this basis, a conclusion was drawn about the usefulness of the PDMAEMA/PVA copolymer for the production of protective fabrics against CWA).

Another elastomer that was used in the QCM sensor to test the sorption of HD simulant was the polyurethane material described in work [67]. The polymer was prepared from poly(tetramethylene oxide) (PTMO) with a molar mass of 2000 g·mol^−1^, hydrogenated methylene diphenyl diisocyanate (HDMI) and 1,4-butanediol (BDO) with 43% hard segments (in the cited work, the material was dubbed 2kPTMO-BDO-HMDI). The material showed elastomeric properties (T_g_ = −76 °C). A variety of quartz microbalance with dissipation analysis, QCM-D, was used for the research. Devices of this type, in addition to changing the resonant frequency while the device is powered, allow to measure the time in which the crystal vibrations disappear after turning the power off. This type of microbalances is used mainly in biological research because the measurement of dissipation allows to determine the structural changes occurring on the crystal surface. In the cited work, QCM-D was used due to the significant swelling of the polyurethane film as a result of sorption of analytes. In the study, the following substances were used as HD simulants: 1-chloropropane, 1-chlorobutane, 1-chloropentane, DCP, diethyl sulfide, and CEES. Research has shown that the polyurethane film sorbs all HD simulants to an almost equal extent. On the basis of the analysis of the properties of the simulants and the magnitude of their sorption, it was concluded that substances with a high dipole moment or strong polarizability are well absorbed in the polymer. At the same time, by comparing the results reported in this work with other results concerning elastomeric layers, it can be stated that the selectivity of this material is rather limited.

### 4.2. Sensor with Layers of Composite Materials

Although elastomers are the most commonly used materials for sensor layers, a number of other materials can also be found in the available literature. Heterogeneous composites constitute a significant group. Depending on the composition, these materials can sorb the analytes on the surface or in the volume of the layers.

**Figure 7:**
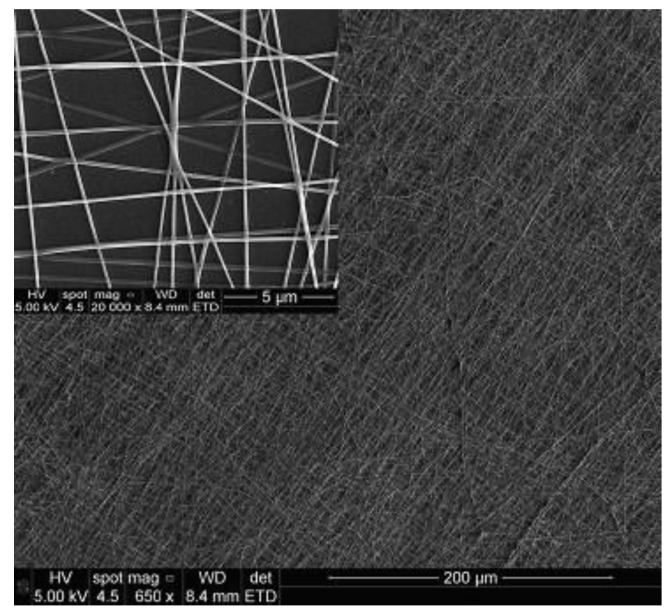
SEM image of the sensor layer made of polyvinyl alcohol (PVA) nanofibers on the SiO_2_ guiding layer of Love wave SAW sensor [68]. Reprinted with permission from [68]. Copyright 2014 Elsevier.

The work [68] describes an array of six Love wave SAW sensors, which was used to detect blister and nerve CWAs simulants. Individual sensors in the array were coated with polymer nanofibers. The nanofibers were used to increase the adsorption surface compared to the traditional polymer film. The electrospinning technique was used to form nanofibers and to apply them on the SAW sensors. In the electrospinning technique, a polymer solution contained in a syringe is applied on the substrate on which the layer is to be formed. A high voltage is applied between the needle tip and the substrate. The electrostatic force pulls the droplets out of the needle and stretches them to form thin streams of polymer solution. During the flight, the solvent evaporates and nanofibers deposit on the substrate. The SEM image of sensor layer made of PVA nanofibers is presented in the Figure 7.

In this work, the authors used nanofiber sensor coatings made of the following materials: polyvinyl alcohol (PVA), polystyrene (PS), poly-vinyl-pyrrolidone (PVP), and composites of PVA and SnCl4, PS, and copolymer poly (styrene-alt-maleic anhydride) (PSMA). The simulants of nitrogen mustard, sulfur mustard, and nerve agents were used to test the array performance: DPGME, DMA, and DMMP, respectively. The array response was analyzed using PCA. Research has shown that the array detected and correctly classified all analytes.

Another work presenting the use of composite material was published in 2018 [69]. As a sorptive layer of a QCM, nanoclay composites (Montmorillonite with particle sizes below 100 nm) based on PECH, alkyd resin, and collodion were used. A PECH-based composite sensor was used for HD detection. As the research showed, a QCM sensor with a center frequency of 10 MHz coated with a nanoclay (12.5%)–PECH layer showed about 2.5 times greater sensitivity towards HD than a sensor with layer made of a pure PECH with the same layer thickness. HD limit of detection was 0.17 ppm. The authors of the study tried to explain such a significant increase in sensitivity due to increase of adjoining space with nanoclay particles.

### 4.3. Sensors with Layers of Oxide Materials

Acoustic wave sensors with oxide layers have also been used to detect various VOCs. The oxide layers usually exhibit semiconductor properties and adsorption of target analyte on their surface changes the conductivity of the layer. As a rule, sensors with this type of layer work at significantly elevated temperatures. The sensor response is generated by means of electrical loading (although there are exceptions to this rule), which in turn limits the application of such layers to sensors with surface acoustic waves (sensors with bulk waves such as QCM are not sensitive to electrical effects).

**Figure 8:**
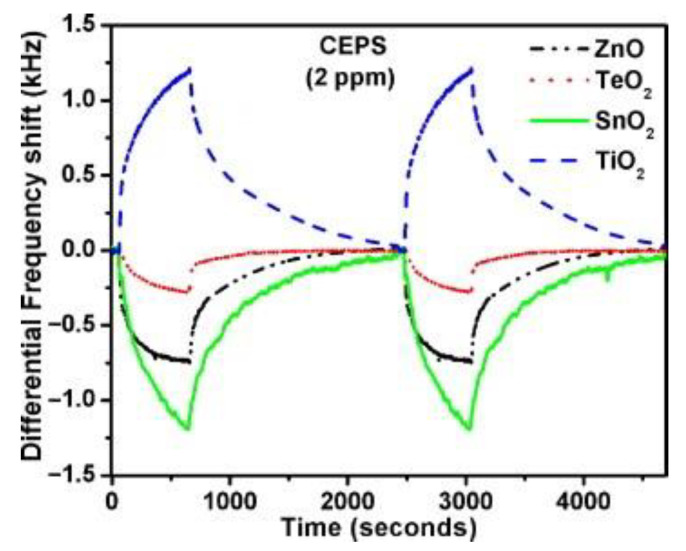
Real-time differential frequency shift ΔF plots of four SAW sensors with oxide layers recorded during exposure to CEPS vapor [71]. ΔF is defined as f_0_-f_s_ (where f_0_, f_s_—frequency before and after exposure to analyte vapors). Reprinted with permission from [71]. Copyright 2013 Elsevier.

In the case of the blister CWAs detection, sensors with oxide layers were also used [70]. A noteworthy study concerning detection of simulants of these substances was published in 2013 [71]. This paper describes an array of four SAW sensors with a center frequency of 433.9 MHz, coated with ZnO, TeO_2_, SnO_2_, and TiO_2_. In this study, the following simulants were used: DBS, CEPS, DMMP, and diethyl chlorophosphate (DECP). The array enabled the detection and classification of individual simulants even in the presence of interferents (petrol, diesel, kerosene, acetone, and water vapor) at room temperature. The analysis of the responses of individual sensors revealed that the adsorption of simulants vapors causes an increase in the resonance frequency of ZnO, TeO_2_, and SnO_2_ sensors and a decrease in the frequency in the case of TiO_2_. Based on additional measurements of changes in the conductivity of the sensor layers due to the sorption of simulants vapors, a significant contribution of the electrical effect to the sensor response was excluded. By deduction, it was found that the response generation mechanism is based mainly on the elastic loading. As a result of vapor sorption, the sensor layers are stiffened (probably by sorption of analytes in the pores and in intergranular spaces) in the case of ZnO, TeO_2_, and SnO_2_ and the resonance frequency increases. The recorded real-time array response to CEPS vapor is shown in Figure 8.

### 4.4. Other Materials and Solutions

The available literature also includes descriptions of sensor that are difficult to classify in terms of the sorptive layers used in any of the above-described groups. 

In the SAW sensor used for the detection of sulfur mustard described in [72], a semiconductor polymer sorptive layer was used. This layer was a combination of palladium phthalocyanine (content 30%) and polyaniline (70%) and was dubbed by authors as PcPd_0.3_PANI_0.7_. The described sensor had a sensitivity of 105 kHz·mg^−1^·m^3^ to sulfur mustard and good dynamic properties (at the HD concentration of 7 mg·m^−3^, the response time constant was approximately 2 min). As stated by the authors of the work, the sensor response was generated by an electrical effect. HD adsorption on the sensor surface caused a decrease in the conductivity of the sensor layer, which resulted in a decrease in the resonance frequency of the SAW device. Despite the authors drawing conclusions about the suitability of the sensor with the PcPd_0.3_PANI_0.7_ layer for HD detection, the work lacked an analysis of the sensor selectivity.

The work [73] presents a QCM sensor with an adsorption layer made of epoxy resin. In the study, diglycidyl ether of bisphenol A and epichlorohydrin monomers were used to prepare epoxy resins. Due to the high T_g_ at the measurement temperature, the sensor material showed a decidedly glassy character. The sensor made with QCM with a center frequency of 10 MHz exhibited good selectivity to HD. The selectivity studies were carried out in the test set: DCEE, carbon tetrachloride, acetone, ethanol, and toluene. The sensors’ sensitivity to HD was approximately three times greater than that of its simulant (DCEE). However, the sensor layer degraded over time and after 15 months it showed a decrease in the HD response by about 40%.

**Figure 9:**
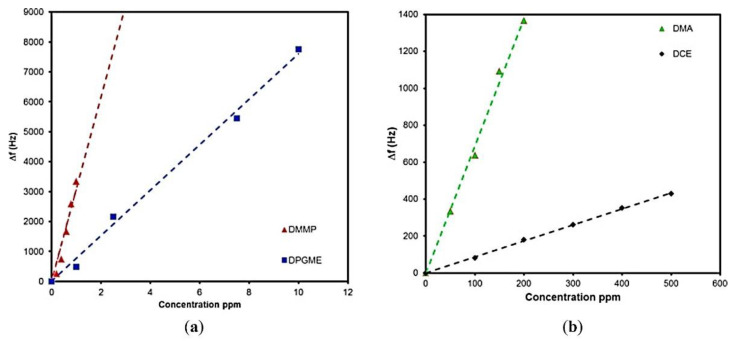
Response versus concentration plots of graphene oxide Love wave sensor for (**a**) DMMP and DPGME, and (**b**) DMA and DCE [74]. Reprinted with permission from [74]. Copyright 2016 Elsevier.

Interesting results also concerning the sensor with an adsorption layer were published in 2016 [74]. In this case, the Love wave sensor (center frequency 164 MHz) with sorptive layer made of graphene oxide (GO) was described. A coating was formed by applying the suspension of graphene oxide in ethanol on the sensor guiding layer. Next, the responses of the sensor to nitrogen mustard simulant (DPGME), sulfur mustard simulants (DCE and DMA), and sarin simulant (DMMP) were investigated. The response versus concentration curves for individual simulants are presented in Figure 9.

The sensor showed the highest sensitivity to DMMP and DPGME (3087 and 760 Hz·ppm^−1^, respectively). The calculated detection limits for these compounds were 9 and 40 ppb, respectively. According to the authors, the adsorption mechanism of GO was based on the formation of acidic hydrogen bonds between the hydroxyl and carboxyl groups of GO and the basic CWA simulants. On the basis of the presented results, it can be concluded that the sensor had excellent static and dynamic parameters and allowed the detection of blister and nerve CWAs at concentrations lower than their threshold toxic concentrations. However, the selectivity of the sensor has not been thoroughly investigated in this study.

**Figure 10:**
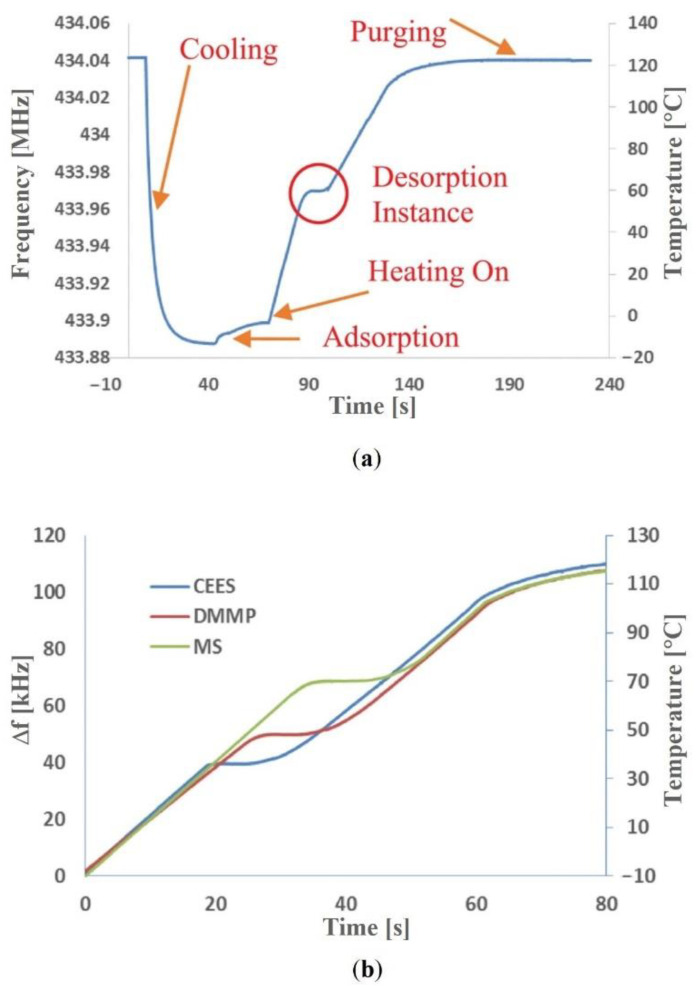
Application of the uncoated SAW sensor with temperature programmed desorption for CWA simulants detection: (**a**) changes in the resonant frequency of the sensor related to the various stages of the device operation; (**b**) the sensor responses resulting from the detection of the CEES, DMMP, and MS [75]. Reprinted with permission from [75]. Copyright 2021 IEEE.

A new approach to the detection of CWA vapors (including blister agents) using SAW sensors was presented in the work [75]. In this case, a measurement system with an uncoated SAW resonator was presented. The detection of CWA simulants was based on temperature programmed desorption. The detection of CWA vapors consisted in cooling the SAW resonator to a temperature of about −10 °C. Then, the element was exposed to the analyzed gas. At this stage, analytes were adsorbed on the surface of the resonator. Then, the resonator was heated to a temperature of about 120 °C with a rate of 2 °C·s^−1^. During this stage, thermal desorption of previously adsorbed substances took place. At the moment of analyte desorption (occurring at a strictly defined temperature), the sensor generated a frequency signal related to the mass loss of the analyte. This simple measurement method allowed for the detection and differentiation of CEES, MS, and DMMP at concentrations of 0.1 ppm. The mechanism of selective detection of substances was based on the differences in their volatility and strength of interaction with the polar quartz substrate of the SAW resonator. In fact, the solution proposed in the study was a combination of a preconcentrator with a thermal desorber and a device for precise mass measurement. Figure 10 shows the changes in the resonant frequency of the sensor related to the successive stages of the device operation (a) and the sensor responses resulting from the detection of the individual analytes (b).

## 5. Discussion and Conclusions

As mentioned in the introduction, the detection of blister agents is also possible with the use of semiconductor and fluorescent/colorimetric sensors. Such sensors are simple, affordable, and have low instrument requirements. For these reasons, they represent a potential alternative to acoustic wave sensors in some applications.

In the case of semiconductor sensors, layers made of materials such as WO_3_, SnO_2_ [18], and Mn_3_O_4_ [19] are used. In such sensors, semiconductor materials are usually modified with various metals, e.g., Au, Pt, Pd, or Eu. When exposed to an atmosphere containing target analytes, the semiconductor layers change their resistance. The change in resistance is measured with a simple conductometric system. However, sensors of this type require operation at a significantly elevated temperature (200–400 °C). Moreover, the literature does not exhaust the issue of their selectivity.

In the case of optical sensors (fluorescent or colorimetric), a reaction takes place that changes the color of the analyte solution or produces luminescence in the range of a specific wavelength. Sensors of this type for the detection of HD and CEES use a nucleophilic attack of the probe molecule on the electrophilic site of analyte to generate fluorescence/colorimetric response [76,77,78,79,80,81]. This idea allows the detection of HD and its simulants at very low concentrations. Moreover, thanks to the use of an appropriate probe molecule, it is possible to obtain high selectivity. Due to the fact that the occurring reaction is usually covalent, the sensor signal is irreversible. In addition, most of the studies described in the literature concern liquid phase analysis, similar to fluorescence sensors for the detection of nerve CWAs [82].

In [83], a fluorescence sensor capable of detecting sulfur mustard vapors (analysis in the gas phase) was described. For the detection of HD and CEES, hierarchical fluorene-based microspheres were used here, which interacted non-covalently (sulfur–π interactions, dipole–dipole interactions) with analyte molecules. The interactions change the fluorescence emission spectrum of the microspheres in a proportional and specific manner for the detected analytes. As the tests revealed, the sensor showed good selectivity and high speed of operation, but the sensor signal was not reversible. 

Similarly, the detection of CEES in the gas phase is described in [78]. However, in this case, the authors only presented the possibility of a qualitative CEES detection in the gas phase, without providing a precise limit of detection or test concentrations.

Table 3 summarizes some types of simple and undemanding sensors that allow the detection of blister agents and their simulants in the gas-phase. This table compares some features of fluorescent/colorimetric and semiconductor sensors with acoustic wave sensors.

Based on the analysis of the table, it can be said that both fluorescent/colorimetric and semiconductor sensors show much lower detection limits than acoustic wave sensors. Acoustic wave sensors, however, are characterized by the reversibility of the analytical signal, short response time and the fact that they operate at room temperature.

Some limitations of acoustic wave sensors are also evident by comparison with well-established analytical techniques. In this case, the LOD of devices based on acoustic wave sensors are in general significantly higher (e.g., HD LOD for commercial M90 by Environics, Mikkeli, Finland (IMS), AP2C by Proengin, Saint-Cyr-l’Ecole, France (FP) and APD2000 by Smiths Detection, London, England (SAW) analyzers are 0.4, 2 and 40 mg·m^−3^, respectively [23,84]).

Another limitation is the degradation of selective polymer coatings of sensors observed in some studies [73] or the relatively low selectivity of a single sensor. In some studies, insufficient selectivity of sensor arrays and susceptibility of devices to interference are also reported [23].

It is also worth paying attention to the fact that despite the simplicity, the operation and reading of the acoustic wave sensor indications requires an external electronic system and power supply. Of course, these requirements are much lower than in the case of classical analytical techniques such as the aforementioned IMS or FP. Nevertheless, in this respect, acoustic wave sensors are much more demanding and complicated than colorimetric sensors, in which the visual reading can be made with the naked eye.

Based on the analysis of the studies presented in this review, it can be seen that the acoustic wave sensor technique makes it possible to detect and identify blister CWAs and their simulants. Although the first works on this subject were published in the 1990s, new selective coatings are still being intensively researched. Analyzing the cited works, it is possible to put forward some general theses about the detection of blister CWAs and their simulants with the use of this technique:

1. First, although selective sensor materials are available, the analytical capabilities of a single sensor are very limited. Considerable cross-sensitivities to different VOCs can cause false readings. This problem is solved by sensor arrays. As it has been shown many times in this review, the sensor array equipped with appropriately selected sorptive layers allows for the correct identification or at least classification of the detected compounds. Various pattern recognition and dimension reduction algorithms are very useful in this process.
2. Detection of blister CWAs at concentrations lower than their threshold toxic concentrations is possible with the use of preconcentration. A preconcentrator with a thermal desorber lowers the sensor detection limit and reduces the influence of interferents by removing them from the analyzed gas stream. The disadvantage, however, is the extension of the analysis time.
3. Information on the degradation of elastomeric layers over time has also been found in a number of studies. This phenomenon can be problematic when implementing gas analyzers for use.

Looking more broadly at the achievements in the field of acoustic wave sensors (chemical sensors of various gases and volatile organic compounds, sensors of other environmental pollutants, biological sensors), it can be said that this technique is now well-established. This is evidenced by a large amount of advanced works in scientific journals, extensive patent literature, and many commercially available solutions (mainly in the case of biological sensors). As a result, transducers with acoustic wave and numerical methods of array responses interpretation have reached a high level of advancement.

In the case of sensors for blister CWAs detection, the main direction of development is still research on coating materials. The emerging new materials and methods of applying layers create opportunities for further development of this technique. An example of promising material class are highly selective molecularly imprinted polymers—MIPs. Materials of this type show high steric compatibility and compatibility of intermolecular interactions with the analyte molecules. The work [85] describes the MIP polymer dedicated to HD sorption. It has been proven that this MIP has a high and selective HD sorption capacity. In the available literature, however, we have not found a description of studies in which such materials would be used in acoustic wave sensors for HD detection (despite the fact that there are studies of acoustic wave sensors with other MIP polymers for the detection of many other chemical substances [86]).

The literature reports presented in this review concern basic research and application of conceptual solutions. Over the years, several complete, commercially available devices for CWA detection (including blister agents) have also been designed. Most of them were handheld analyzers equipped with SAW sensor arrays with elastomeric coatings (although manufacturers usually do not disclose exactly what polymers were used in their composition). These devices are also equipped with preconcentrators. Among these analyzers, we can list: SAW MiniCAD by MSA, Cranberry Township, PA, USA (detects HD at concentrations of 1 mg·m^−3^ [87]), JCAD (detects HD at a concentration of 40 mg·m^−3^ in 8 s but is susceptible to influence of interferents, e.g., aliphatic halogens [23]), and JCAD ChemSentry 150C by BAE Systems, Farnborough, England. Currently, there are also available devices using SAW sensor arrays combined with an additional detection technique, e.g., electrochemical sensors (Hazmatcad, Hazmatcad Plus by MSA, Cranberry Township, PA, USA and CW Sentry 3G by Arrow-Tech Inc., Rolla, ND, USA) [88]. In the review, however, we did not devote much space to the description of these devices, because in most cases, their metrological parameters were not confirmed by studies published in the peer-reviewed scientific literature.

## Figures and Tables

**Table 1:** Some physicochemical properties and toxicity of HD and its simulants (LD_50_ stands for Lethal Dose 50; LD_Lo_ stands for Lethal Dose Low).

CWA/Simulant	Systematic Name/CAS	Saturated Vapor Pressure (mmHg) at 25 °C	LogK_ow_	LD_50_ Rat Oral (mg·kg^−1^)	LD_50_ or LD_Lo_ Mouse Intraperitoneal (mg·kg^−1^)
HD Sulfur Mustard	bis(2-chloroethyl) sulfide/505-60-2	0.11	2.41–2.55	2.4 [38]	4.8 [30]
CEES/HM Half Mustard	2-chloroethyl ethyl sulfide/693-07-2	3.4	2.2	252 [39]	17.7 [30]
DCP	1,5-dichloropentane	1.1	3.3	-	64 [40]

**Table 2:** LSER coefficients of poly(epichlorohydrin) (PECH) determined at temperature of 25 °C.

	Constant (c)	Polarizability (r)	Dipolarity/ Polarizability (s)	Hydrogen Bond Basicity (a)	Hydrogen Bond Acidity (b)	Dispersion and Cavity (l)
PECH	−0.75	0.10	1.63	1.45	0.71	0.83

**Table 3:** Comparison of different types of sensors enabling the detection of blister agents and their simulants in the gas phase.

Type of Sensor	Selective Material	Target Analyte	LOD [ppb]	Response Time	Operating Temperature [°C]	Response Reversibility	Literature
Fluorescent/colorimetric	fluorene -based hierarchical microspheres	HD	30	single seconds	RT *	no	[83]
Semiconductor	Au-popped Mn_3_O_4_	DPGME	0.6	single minutes	200–300	yes	[18]
Love-wave SAW	graphene oxide	DPGME	40	single minutes	RT	yes	[74]
Rayleigh-wave SAW	PECH	CEES	120	several seconds	RT	yes	[55]

* RT—room temperature.

## Data Availability

The data presented in this study are available on request from the corresponding author.
